# Phlorotannins with Potential Anti-Tyrosinase and Antioxidant Activity Isolated from the Marine Seaweed *Ecklonia stolonifera*

**DOI:** 10.3390/antiox8080240

**Published:** 2019-07-24

**Authors:** Bandana Manandhar, Aditi Wagle, Su Hui Seong, Pradeep Paudel, Hyeung-Rak Kim, Hyun Ah Jung, Jae Sue Choi

**Affiliations:** 1 Department of Food and Life Science, Pukyong National University, Busan 48513, Korea; 2 Department of Food Science and Human Nutrition, Chonbuk National University, Jeonju 54896, Korea

**Keywords:** mushroom tyrosinase, 974-A, Western blot analysis, phlorotannins, antioxidant, molecular docking simulation

## Abstract

Compounds were isolated from *Ecklonia stolonifera* Okamura, a marine brown alga widely consumed as food. Among the isolated compounds, 974-A was demonstrated for the first time to be a potent competitive inhibitor of mushroom tyrosinase activity towards l-tyrosine and l-DOPA (IC_50_ values = 1.57 ± 0.08 and 3.56 ± 0.22 µM, respectively). Molecular docking simulations clarified that the hydroxyl residues of the isolated compounds formed hydrogen bonds with residues at the catalytic and allosteric sites of tyrosinase, while other residues participated in hydrophobic interactions. Moreover, 974-A, phlorofucofuroeckol-A and eckol reduced the cellular melanin content and tyrosinase activity, and downregulated the expression of melanogenesis enzymes including tyrosinase, tyrosinase-related protein (TRP)-1, and TRP-2 in B16F10 melanoma cells. These compounds also effectively scavenged radicals at the cellular level. Thus, our results revealed that compounds isolated from *E. stolonifera* are potent tyrosinase inhibitors with potential applications in the cosmetic industry for treatment of hyperpigmentation and for the anti-browning effect in the agricultural field.

## 1. Introduction

Tyrosinase has become an enzyme of concern due to its roles in both the melanogenesis of mammals and the enzymatic browning of fruits and fungi. Upon exposure to UV radiation, melanocytes synthesize melanin, which protects nuclear DNA from UV-induced damage and binds reactive oxygen species (ROS) [1]. Melanogenesis is initiated with the oxidation of tyrosine to dopaquinone, which is catalyzed by tyrosinase (TYR). This is the rate-limiting step in melanin synthesis, as the subsequent reactions can proceed spontaneously at physiological pH values [2]. Furthermore, dopaquinone is converted to l-DOPA and l-dopachrome through an autooxidation reaction in which l-DOPA, the substrate of tyrosinase, is oxidized to dopaquinone again by tyrosinase.

Tyrosinase-related protein-1 (TRP-1) and TRP-2 or dopachrome tautomerase also play an imperative part in all eumelanin-yielding reactions. TRP-1 has been proposed to increase the eumelanin: pheomelanin ratio [3], whereas TRP-2 mediates the tautomerization of l-dopachrome (a red melanin precursor) to the colorless dihydroxyindole-2-carboxylic acid (DHICA). In the absence of TRP-2, l-dopachrome is spontaneously converted to the toxic melanin precursor, dihydroxyindole (DHI). Thus, TRP-2 may protect melanocytes against cytotoxicity by limiting DHI formation [4]. On the other hand, TRP-1 also protects against oxidative stress via the peroxidase effect [3].

Hence, tyrosinase, along with other melanocyte-specific markers, is important for tyrosinase inhibitory studies. A series of enzymatic and non-enzymatic reactions ultimately form eumelanin and pheomelanin pigments. In addition to eumelanin and pheomelanin, another “melanin” that relies on phenolic monomers other than tyrosine is termed as allomelanin.

The browning phenomenon in fruit and fungi is also usually related to oxidative polymerization, which is theoretically similar to melanogenesis [5]. Thus, there is an ongoing search for novel tyrosinase inhibitors for cosmetic and food applications, as hyperpigmentation in humans and enzymatic browning in food are undesirable, and tyrosinase is a major reason for these conditions. Tyrosinase inhibitors from natural sources have had praiseworthy success in recent years because they exhibit a plethora of benefits over synthetic and semi-synthetic products.

Overexposure to sunlight induces the overproduction of reactive oxygen species (ROS)/free radicals, which damage lipids, proteins, and deoxyribonucleic acids. Excess sun exposure also increases the expression of collagenase, a protease that degrades collagen, which can result in photoaging and wrinkling of the skin [6]. ROS/free radicals also play an important role in the biosynthesis of melanin. Several studies have demonstrated that the catalytic reactions of tyrosinase that generate dopaquinone require free radicals, which can undergo various oxidation reactions, thus increasing the production of melanin [7] during melanogenesis. Hence, compounds with good antioxidant potential and anti-tyrosinase activity could ameliorate hyperpigmentation disorders and prevent the browning of food products.

Due to their numerous biological and phytochemical benefits, marine plants have become important nutraceuticals and pharmaceuticals. *Ecklonia stolonifera* Okamura (*E. stolonifera*), the representative perennial brown alga among *Ecklonia* species, is a member of the family Laminariaceae, belonging to the order Laminariales [8]. It is usually found in subtidal zones at a depth of 2–10 m, and is widely distributed along the eastern and southern coasts of Korea [9]. This seaweed is frequently used as food, along with *Laminaria japonica* and *Undaria pinnatifida*. Several compounds have been isolated from these marine seaweeds, with phlorotannins being the major class. Numerous biological activities of phlorotannins have been reported, including antioxidant [8], anti-inflammatory [10], neuroprotective [11], anti-diabetic [12], anti-hypertensive [13], hepatoprotective [14], anti-microbial [15], anti-photoaging [16], and anti-tyrosinase activities [17]. Thus, in the present study, we examined the potential of *E. stolonifera*-derived compounds to prevent hyperpigmentation through a bio-assay-guided evaluation of mushroom tyrosinase inhibition. We also investigated their antioxidant potential and intracellular ROS scavenging abilities, performed a molecular docking simulation, and assessed their effects on cellular signaling through Western blot analysis to understand the underlying mechanisms.

## 2. Materials and Methods

### 2.1. Chemicals and Reagents

l-Tyrosine, l-DOPA, mushroom tyrosinase (EC 1.14.18.1), kojic acid, arbutin, Trolox, l-ascorbic acid, l-penicillamine, diethylene triamine penta-acetic acid (DTPA), 2′,7′-dichlorofluorescein diacetate (DCFH-DA), dihydrorhodamine (DHR) 123, 2,2′-azino-bis (3-ethylbenzothiazoline-6-sulphonic acid) (ABTS^•+^), potassium persulfate, *tert*-butyl hydroperoxide (*t*-BHP), 2,2-diphenyl-1-picrylhydrazyl (DPPH), dimethyl sulfoxide (DMSO), 3-(4,5-dimethylthiazol-2-yl)-2,5-diphenyltetrazolium bromide (MTT), and radioimmunoprecipitation assay (RIPA) buffer were purchased from Sigma Aldrich (St. Louis, MO, USA). Dipotassium phosphate was obtained from Junsei Chemical Co. Ltd. (Tokyo, Japan), and monopotassium phosphate was obtained from Yakuri Pure Chemicals Co. Ltd. (Osaka, Japan). Peroxynitrite (ONOO^−^) was obtained from Calbiochem, Merck Millipore (Burlington, MA, USA). Dulbecco’s modified Eagle’s medium (DMEM) was purchased from Mediatech Inc., a Corning subsidiary (Manassas, VA, USA). Penicillin-streptomycin, 0.25% trypsin-ethylenediaminetetraacetic acid (EDTA), and fetal bovine serum (FBS) were bought from Gibco BRL Life Technologies (Grand Island, NY, USA). Alpha-melanocyte-stimulating hormone (α-MSH) was purchased from Alomone Labs (Jerusalem, Israel). An enhanced chemiluminescence (ECL) detection kit was procured from Thermo Scientific (Rockford, IL, USA). The primary antibodies for tyrosinase, TRP-1 and TRP-2, as well as horseradish peroxidase (HRP)-conjugated anti-mouse IgG, were obtained from Santa Cruz Biotechnology, Inc. (Santa Cruz, CA, USA). All other chemicals and solvents were purchased from E. Merck, Fluka, and Sigma-Aldrich unless otherwise stated.

### 2.2. Plant Material

Leafy thalli of *E. stolonifera* (500 g) were collected from the coast of Busan (Republic of Korea) in August of 2015, and a voucher specimen was deposited in the laboratory of Prof. J. S. Choi. After being washed with seawater and tap water, the samples were air-dried, ground, and stored at −20 °C until use.

### 2.3. Experimental Methods

#### 2.3.1. Extraction and Fractionation

The dried leafy thalli of *E. stolonifera* were refluxed 5 times with 70% ethanol (EtOH) for 3 h. The total filtrate was then concentrated to dryness in vacuum at 40 °C to yield a 70% ethanol extract (200 g). This extract was then suspended in water (H_2_O) and successively partitioned with dichloromethane (CH_2_Cl_2_) and ethyl acetate (EtOAc) to yield CH_2_Cl_2_ (53.3 g), EtOAc (63.8 g), and final residue H_2_O (79.8 g) fractions.

#### 2.3.2. Isolation of Compounds

**Figure 1:**
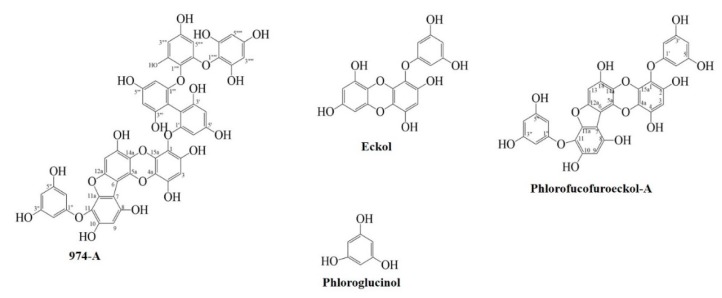
Structure of compounds isolated from *Ecklonia stolonifera.*

The EtOAc fraction (63.8 g) was chromatographed on a Si gel column with an EtOAc: MeOH (30:1 to 5:1, gradient) solvent system to yield 20 sub-fractions (Fr. 1–20). Fraction 3 (2.0 g) was further chromatographed on a Sephadex LH-20 with 100% methanol as the solvent, producing a total of 10 sub-fractions. Among them, sub-fraction 2 (1 g) was chromatographed on a Si gel column with CH_2_Cl_2_: EtOAc: MeOH (7:1:0.1) to yield phloroglucinol (40.0 mg) (sub fraction 10). Sub-fraction number 5 (750 mg) (from Sephadex LH-20 column) was further chromatographed (CH_2_Cl_2_: EtOAc: MeOH (5:1:0.1)) to yield phlorofucofuroeckol-A (40.0 mg) (sub fraction 2) and eckol (33.0 mg) (sub fraction 3). Further, sub fraction number 7 (400.0 mg) (from Sephadex LH-20 column) was chromatographed on a Si gel column with CH_2_Cl_2_: EtOAc: MeOH (3:1:0.1) as the eluent, yielding 8 sub-fractions. The 4th sub-fraction (80.3 mg) was again chromatographed through CH_2_Cl_2_: EtOAc: MeOH (3:1:0.1) to produce 10 sub-fractions, among which sub-fraction 4 (42.0 mg) was further chromatographed through Sephadex LH-20 with 100% methanol as the solvent. Seven sub-fractions were collected, among which sub-fraction 3 was obtained as 974-A (24.5 mg). The structures of the isolated compounds are shown in Figure 1. Compound 974-A was identified by spectroscopic methods including ^1^H- and ^13^C-NMR, and by comparison with published spectral data [18]. Furthermore, phlorofucofuroeckol-A, eckol, and phloroglucinol were isolated from the EtOAc fraction and identified by comparison with published spectral data [12,18].

#### 2.3.3. In vitro Mushroom Tyrosinase Inhibitory Assay

Mushroom tyrosinase inhibitory activity was assessed by the method described by Kang et al. 2004 [17] with slight modifications. Kojic acid and arbutin were used as positive controls.

#### 2.3.4. DPPH Radical Scavenging Activity

DPPH radical scavenging activity was evaluated by the method described by Blois, 1958 [19] with slight modifications. l-Ascorbic acid was used as a positive control.

#### 2.3.5. ONOO^−^ Radical Scavenging Assay

ONOO^−^ scavenging activity was assessed by a modified version of Kooy’s method [20], which monitors the production of fluorescent rhodamine 123 from non-fluorescent DHR 123 in the presence of ONOO^−^. l-Penicillamine was used as a positive control.

#### 2.3.6. ABTS^•+^ Radical Scavenging Assay

This assay assesses the ability of different substances to scavenge ABTS radical cations (ABTS^•+^) in comparison with the positive control, Trolox. The method was developed by Re et al. 1999 [21].

#### 2.3.7. Kinetic Parameters of Mushroom Tyrosinase Inhibition

The inhibition mode and inhibition constant (*K_i_*) for mushroom tyrosinase inhibition were calculated from the Lineweaver-Burk and Dixon plots [22,23]. Kinetic parameters were obtained for various concentrations of the substrates (l-tyrosine and l-DOPA) and inhibitors (974-A, phlorofucofuroeckol-A and eckol). Graphs were generated with Sigma plot 12.0 (SPSS Inc., Chicago, IL, USA).

#### 2.3.8. Mushroom Tyrosinase Molecular Docking Simulation

Molecular docking analysis was carried out by a previously reported procedure [24]. The structures of 974-A were obtained from the PubChem Compound (NCBI) database and converted to 3D structures by Marvin Sketch (v17.1.30, ChemAxon, Budapest, Hungary). AutoDock 4.2 was used for docking simulations, and grid maps were generated in the Autogrid program. The docking protocol for rigid and flexible ligand docking comprised 20 independent genetic algorithms. Docking results were visualized and analyzed with PyMOL (v1.7.4, Schrödinger, LLC, Cambridge, MA, USA) and Discovery Studio (v16.1, Accelrys, San Diego, CA, USA).

#### 2.3.9. Cell Culture and Viability Assay

B16F10 mouse melanoma cells were obtained from the American Type Culture Collection (Manassas, VA, USA). Cells were cultured at 37 °C in high-glucose DMEM supplemented with 10% FBS, 100 U/mL penicillin, and 100 μg/mL streptomycin in a humidified atmosphere containing 5% CO_2_. The MTT assay was used to determine the cytotoxicity of 974-A, phlorofucofuroeckol-A, and eckol according to previously described methods [24] with slight modifications. Briefly, B16F10 cells were seeded in a 96-well plate at 1 × 10^4^ cells/well and incubated for 24 h in DMEM supplemented with 10% FBS. Then, the culture medium was replaced with FBS-free DMEM, and the cells were treated with different concentrations of compounds (6.25, 12.5, 25 and 50 μM) with or without α-MSH (3 μM) for 24 h. After 24-h incubation of the samples, the FBS-free DMEM was suctioned, and the cells were treated with an MTT solution (100 μL at 0.5 mg/mL in phosphate-buffered saline (PBS)) and incubated for 2 h. The proportion of surviving cells was determined after the medium was replaced with 100 µL of DMSO. Control cells were treated with 0.1% DMSO, as DMSO exhibited no cytotoxicity at this concentration in this assay. The absorbance was measured at 570 nm on a microplate reader (Molecular Devices, Sunnyvale, CA, USA).

#### 2.3.10. Melanin Content Assay

The intracellular melanin content was determined according to a procedure described previously [25] with minor modifications. Briefly, B16F10 cells were seeded in a 24-well plate at 1 × 10^4^ cells/well and incubated for 24 h in DMEM containing 10% FBS. Cells were pretreated with various concentrations of 974-A, phlorofucofuroeckol-A, and eckol for 1 h, and then were stimulated with α-MSH (3 μM) for 24 h. The cells were washed with PBS, and were dissolved in 1 N NaOH containing 10% DMSO by being boiled at 80 °C for 30 min. The cell lysates were centrifuged at 14,000 rpm for 10 min, and the absorbance of the supernatant was measured at 405 nm on a microplate reader (Molecular Devices). The absorbance was normalized to the total protein content for the determination of the melanin content. Arbutin (500 μM) was used as a positive control.

#### 2.3.11. Cellular TYR Activity Assay

Cellular TYR activity was measured as l-DOPA oxidase activity by a previously described method [26] with some modifications. Cells were seeded at 1 × 10^4^ cells/well in a 24-well plate, incubated for 24 h and then stimulated with α-MSH (3 μM) in the presence or absence of 974-A, phlorofucofuroeckol-A, or eckol for 24 h. After treatment, the cells were washed with cold PBS and lysed with RIPA buffer. The cell lysates were centrifuged at 12,000 rpm for 10 min, and the supernatant was used as the cellular TYR solution. The reaction mixture, which contained 80 μL of the cell lysate and 20 μL of l-DOPA (20 mM), was incubated at 37 °C for 45 min. After the incubation, dopachrome formation was measured spectrophotometrically every 10 min for 45 min at 450 nm on a microplate reader (Molecular Devices). TYR activity was calculated as a percentage of the control. Arbutin (500 μM) was used as a positive control.

#### 2.3.12. Determination of the Intracellular ROS Level

Intracellular ROS production was assessed with DCFH-DA, an oxidant-sensitive fluorescent probe. B16F10 melanoma cells were seeded in black 96-well plates at 1 × 10^4^ cells/well and incubated with various concentrations (12.5, 25, 50 or 100 μM) of 974-A, phlorofucofuroeckol-A, and eckol for 24 h. The cells were then exposed to *t*-BHP (400 μM) for 2 h to induce ROS production, and were subsequently incubated with DCFH-DA (25 μM) for 30 min. The resultant fluorescence intensities were measured at an excitation wavelength of 485 nm and an emission wavelength of 530 nm on a fluorescence microplate reader (FLx 800, Bio-Tek Instruments Inc., Winooski, UT, USA). Trolox (2 mM) was used as a positive control.

#### 2.3.13. Western Blot Analysis

B16F10 cells were stimulated with α-MSH in the presence or absence of compounds for the indicated times. The cells were then washed with ice-cold PBS and lysed with RIPA buffer on ice for 30 min. After centrifugation of the samples at 12,000× *g* for 20 min, the protein concentrations of the supernatants were determined. Aliquots of protein were resolved by sodium dodecyl sulfate-polyacrylamide gel electrophoresis (SDS-PAGE) and transferred onto a nitrocellulose membrane. The membrane was washed with Tris-buffered saline (TBS; 10 mM Tris-HCl, 150 mM NaCl, pH 7.5) supplemented with 0.05% Tween 20 (TBST), blocked with 5% skim milk (w/v) in TBST for 2 h and incubated for approximately 20 h with the primary antibodies. The immunoblots were then incubated with the appropriate secondary antibody coupled to horseradish peroxidase for 2 h, and protein levels were detected with an ECL detection kit. Densitometric analysis of data from at least three independent experiments was performed with a cooled CCD camera system and CS analyzer version 3.00 software. Fold-increases in protein expression were calculated versus normal controls. The experiments were repeated three times for each experimental condition.

### 2.4. Statistical Analysis

Statistical significance was analyzed by Student’s *t*-test in Microsoft Excel 2016 (Microsoft Corporation, Redmond, WA, USA) and was noted at *p* < 0.05, *p* < 0.01 and *p* < 0.001. All experiments were carried out in triplicate, repeated on three separate days, and expressed as the mean ± standard deviation (SD) (n = 3).

## 3. Results and Discussion

### 3.1. Inhibitory Activities of Compounds Isolated from E. stolonifera against Mushroom Tyrosinase

In the present study, we successfully isolated and determined the structure of a novel phlorotannin from *E. stolonifera*, 974-A, along with other known compounds (phlorofucofuroeckol-A, eckol and phloroglucinol) (Figure 1). Compound 974-A was first isolated from *Ecklonia kurome*, and was found to exert antioxidant activity [18]. In our study, 974-A exhibited potent concentration-dependent tyrosinase inhibitory activity, competitively inhibiting both l-tyrosine (IC_50_ = 1.57 ± 0.08 μM) and l-DOPA (IC_50_ = 3.56 ± 0.22 μM) as substrates.

The inhibitory activity of 974-A was followed by those of phlorofucofuroeckol-A and eckol. Although previous reports [17,27] have described lower activity levels of these compounds against tyrosinase, this research revealed that both compounds potently and dose-dependently inhibited tyrosinase (IC_50_ = 3.42 ± 0.01 and 9.12 ± 0.36 μM for l-tyrosine and 8.14 ± 0.29 and 29.59 ± 0.48 μM for l-DOPA, respectively) (Table 1 and Figure S1). This discrepancy with previous findings may have been due to the laboratory conditions in which the compounds were isolated, the environments in which they were stored or the experimental procedures by which they were tested. Moreover, the previous investigators only determined the inhibitory activity with l-tyrosine as a substrate, while we evaluated the activity with both l-tyrosine and l-DOPA as substrates. It is possible that these compounds were not active against one substrate but were active against the other. Hence, evaluating both substrates may have been advantageous for identifying the inhibitory activities of the tested compounds. No significant inhibition of either substrate by phloroglucinol was revealed.

### 3.2. Radical Scavenging Activities of Compounds Isolated from E. stolonifera

The compounds isolated from *E. stolonifera* were evaluated for their radical scavenging ability as a measure of their antioxidant potential. These compounds are polyphenols, and the phenolic hydroxyl number is known to be significantly related to the antioxidant activity of phlorotannins. A higher phenolic hydroxyl number allows more hydrogen atoms to combine with free radicals. This has a strong denounced electronic effect, resulting in the free radical reaction and a greater extent of hydrogen bonding [28]. Among the isolated compounds, 974-A exhibited the most potent radical scavenging activity against ONOO^−^ and DPPH, with IC_50_ values of 0.26 ± 0.06 μM and 0.92 ± 0.11 μM, respectively, followed by eckol and phlorofucofuroeckol-A (Table 1). Eckol was the most potent compound against ABTS^•+^ (IC_50_ = 5.01 ± 0.19 μM), followed by 974-A (IC_50_ 6.29 ± 0.34 μM). l-Penicillamine, l-ascorbic acid, and Trolox were used as positive controls for the ONOO^−^, DPPH, and ABTS^•+^ radical scavenging assays, respectively.

### 3.3. Enzyme Kinetic Analysis of Compounds Isolated from E. stolonifera against Mushroom Tyrosinase

The inhibitory properties of the active compounds were demonstrated with a double-reciprocal Lineweaver-Burk and Dixon plots. Compound 974-A exhibited a competitive mode of inhibition with an increased *K_m_* (Michaelis menten constant), an unchanged V_max_ (maximal rate) with the increase in concentration, while eckol and phlorofucofuroeckol-A exhibited a non-competitive mode of inhibition where *V_max_* decreased, while the *K_m_* remained unchanged with the increase in concentration for both substrates (l-tyrosine and l-DOPA) (Figure S2). The *K_i_* values for l-tyrosine and l-DOPA are shown in Table 1 and Figure S3, which demonstrate that the lower the *K_i_* value, the tighter is the binding affinity with the enzyme and the more effective is the inhibitor. Moreover, the results were in accordance with the molecular docking results where 974-A is a competitive inhibitor, bound to the active site, whereas eckol and phlorofucofuroeckol-A are non-competitive inhibitors, bound to the allosteric site.

### 3.4. Molecular Docking Simulation on Mushroom Tyrosinase

To clarify the binding sites and determine the underlying mechanisms by which the isolated compounds inhibited mushroom tyrosinase, we performed an in silico docking study. The results of the molecular docking simulation are summarized in Table 2. The primary requirement for being an alternative tyrosinase substrate seemed to be the presence of a hydroxylic group and an electron donor group in the phenol ring [29]. All the compounds were able to justify this sentence structurally, with most of their hydroxyl residues forming hydrogen bonds with tyrosinase residues.

**Figure 2:**
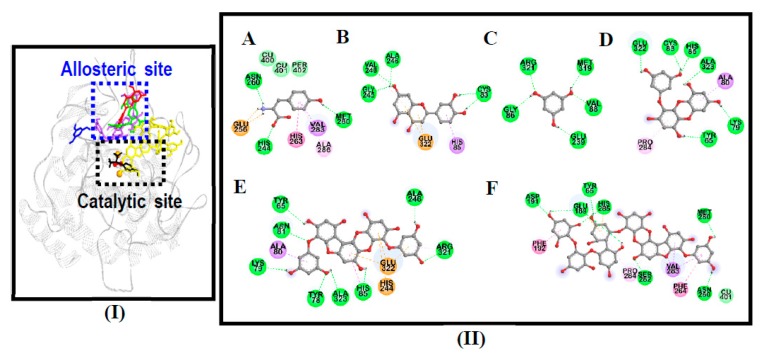
(**I**) Top binding mode of phlorotannins phloroglucinol (blue stick), eckol (green stick), phlorofucofuroeckol-A (purple stick), and 974-A (yellow stick) for the oxy-form tyrosinase with positive controls, l-tyrosine (black stick) and luteolin (red stick) (**A**). Copper and peroxide ions are shown in orange and red spheres, respectively. (**II**) 2D diagram showing l-tyrosine (**A**), luteolin (**B**), phloroglucinol (**C**), eckol (**D**), phlorofucofuroeckol-A (**E**), and 974-A (**F**) binding pose in active site of tyrosinase.

As shown in Figure 2, 974-A displayed a van der Waals interaction at the catalytic binuclear copper center with the Cu401 ion. Furthermore, 974-A (His285, Glu189) exhibited an electrostatic bond, further improving the binding characteristics at the catalytic site. Hydrophobic interactions (amide-π stacked, π-alkyl and π-σ) also stabilized the protein-ligand interaction and positioned 974-A in the catalytic site. However, 974-A displayed a lower binding energy (−3.92 kcal/mol) than the other compounds, possibly due to the heavy structure of the molecule (molecular weight of 974 g/mol); thus, it appears that the binding energy and the inhibitory potency are not always proportional. The relationship between the molecular weights and binding energies of compounds requires further research and interpretation.

Phloroglucinol (−4.87 kcal/mol) interacted at neither the catalytic nor the allosteric site, but displayed five hydrogen bond interactions in the absence of hydrophobic interactions. As shown in Figure 2-I, phloroglucinol had H-bond interactions far from the active (catalytic and allosteric) sites of the enzyme. This is a probable reason for the lack of activity of phloroglucinol against tyrosinase. Eckol displayed six hydrogen bond interactions, whereas phlorofucofuroeckol-A displayed eight hydrogen bond interactions.

Hydrophobic interactions are important for stabilizing the protein-ligand interaction and for positioning compounds in the catalytic and allosteric pockets to inhibit the activity of tyrosinase. In this study, π–σ and π–alkyl interactions were observed between tyrosinase residues and the compounds, and a π–π T-shaped interaction was found between phlorofucofuroeckol-A and tyrosinase residues. Interestingly, electrostatic bonds at histidine and glutamine residues were also found to stabilize the enzyme-phlorofucofuroeckol-A complex. l-tyrosine (a competitive inhibitor, −6.31 kcal/mol) and luteolin (an allosteric inhibitor, −6.19 kcal/mol) were used as standards in the docking trials (Figure 2).

### 3.5. Effect on Cell Viability

An MTT assay was used to determine the cytotoxicity of 974-A, phlorofucofuroeckol-A and eckol towards B16F10 melanoma cells. As shown in Figure S4, five different concentrations (6.25, 12.5, 25, 50, and 100 μM) of the compounds were tested with or without α-MSH co-treatment, and none of them exhibited significant cytotoxicity up to 100 μM.

### 3.6. Effect on α-MSH-Induced Melanin Synthesis and Cellular TYR Activity

**Figure 3:**
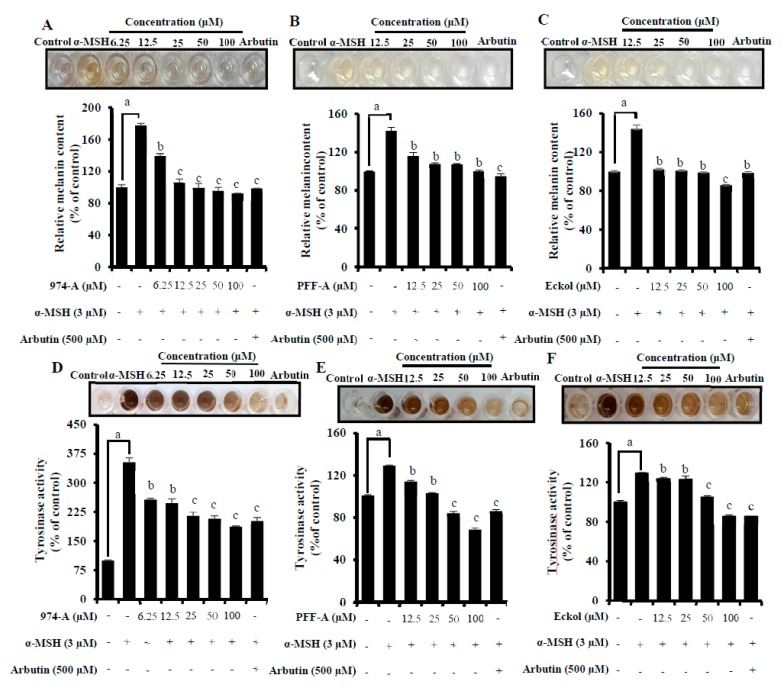
Effects of various concentrations of 974-A (**A**,**D**), phlorofucofuroeckol-A (PFF-A) (**B**,**E**), and eckol (**C**,**F**) on the melanin content and tyrosinase activity, respectively, of α-MSH treated B16F10 melanoma cells. Cells were pretreated with the indicated concentrations (6.25, 12.5, 25, 50, and 100 μM) of 974-A, PFF-A, and eckol for 1h followed by exposure to α-MSH (3.0 μM) for 24h in the presence or absence of the test compound. Arbutin (500 μM) was used as a positive control. Data represent the mean ± SD of three independent experiments. ^a^ *p* < 0.01 indicates significant differences from the normal control group (no treatment); ^b^ *p* < 0.05, ^c^ *p* < 0.01, and ^d^ *p* < 0.001 indicate significant differences from the α-MSH treated group.

It is known that α-MSH related peptides affect the morphology of melanocytes and increase their tyrosinase activity and melanin content by activating their receptors to induce melanogenesis [30]. To examine the effects of 974-A, phlorofucofuroeckol-A, and eckol on melanin production, we treated B16F10 cells with these compounds at concentrations of 6.25-100 μM in the presence of α-MSH, and measured the melanin content after 24 h of treatment. As shown in Figure 3A–C, the melanin content of B16F10 cells was significantly greater after α-MSH treatment than after no treatment. Pretreatment with various concentrations of the isolated compounds remarkably and concentration-dependently attenuated the melanin content of α-MSH-stimulated B16F10 cells. Eckol at 100 μM was more effective than arbutin (500 μM), while 974-A and phlorofucofuroeckol-A at 100 μM were comparable to arbutin in their inhibition. 

To investigate the mechanisms underlying the inhibition of melanogenesis, we measured the TYR activity in α-MSH-stimulated B16F10 cells. After 24 h of treatment with the sample/positive control, the cellular TYR activity of the α-MSH-stimulated B16F10 cells was radically greater than that of untreated cells. On the other hand, the cellular tyrosinase activity of α-MSH-stimulated B16F10 cells pretreated with various concentrations of the compounds decreased in a concentration-dependent manner (Figure 3D–F). These compounds at 100 μM were as active (eckol and 974-A) or more potent (phlorofucofuroeckol-A) inhibitors of cellular tyrosinase than the positive control, arbutin (500 μM). In a previous study, eckol was reported to dose-dependently reduce melanin levels and inhibit tyrosinase at a cellular level [31], comparable to the results we obtained regarding eckol. Also, in a similar study, the phlorotannin 7-phloreckol significantly and dose-dependently reduced the melanin content of IBMX-treated cells [32]. Dieckol displayed 88.9% tyrosinase inhibitory activity, even at 50 μM, and was a stronger inhibitor than kojic acid, a commercial whitening agent. It was also a stronger inhibitor of melanin synthesis than other phlorotannins, although it was weaker than the commercial whitening agent retinol (75.3% at 250 μM) [33]. Hence, it can be concluded that phlorotannins are capable of reducing melanin levels and tyrosinase activity. The inhibitory activities of the phlorotannins 974-A and phlorofucofuroeckol-A towards melanin synthesis and tyrosinase activity in α-MSH-treated cells were evaluated for the first time in this study.

### 3.7. Effect on the Expression of Melanogenic Enzymes

**Figure 4:**
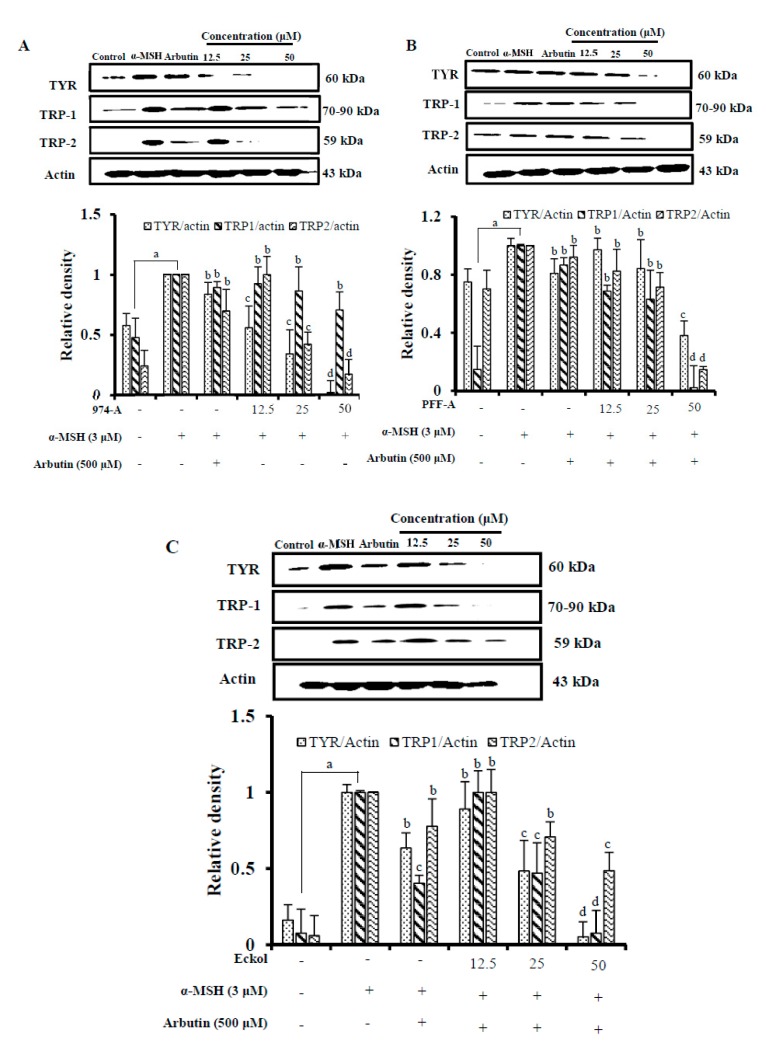
Effect of different concentrations of 974-A (**A**), phlorofucofuroeckol-A (PFF-A) (**B**), and eckol (**C**) on tyrosinase (TYR), tyrosinase related protein-1 (TRP-1), and TRP-2 expression level in α-MSH. Cells were pretreated with the indicated concentration of 974-A, phlorofucofuroeckol-A (PFF-A), eckol along with α-MSH and arbutin for 24 h. Western blotting was performed and protein band intensities were quantified by densitometric analysis using CS analyzer Eng software. Upper panels display representative blots. Equal protein loading was ensured and normalized against β-actin levels. Data represent the mean ± SD of three independent experiments. ^a^ *p* < 0.01 indicates significant differences from the normal control group (no treatment); ^b^ *p* < 0.05, ^c^ *p* < 0.01, and ^d^ *p* < 0.001 indicate significant differences from the α-MSH treated group.

In mammalian melanocytes, melanogenesis is controlled by at least three regulatory proteins: tyrosinase, TRP-1, and TRP-2. To assess the expression of these melanogenic proteins, we performed Western blot analysis on whole-cell lysates of B16F10 cells that had been stimulated with α-MSH and treated with 974-A, eckol, and phlorofucofuroeckol-A. As shown in Figure 4A, treatment with 974-A (12.5–50 μM) dose-dependently attenuated the expression of tyrosinase, TRP-1, and TRP-2, although it downregulated TYR and TRP-2 more effectively than TRP-1. On the other hand, phlorofucofuroeckol-A and eckol (12.5–50 μM) dose-dependently attenuated the expression of TYR, TRP-1, and TRP-2, and reduced the expression of all three proteins to similar degrees (Figure 4B,C). All the compounds downregulated the expression of these proteins more effectively than arbutin (500 μM) when applied at concentrations as high as 50 μM. Hence, these phlorotannins may attenuate α-MSH-stimulated hyperpigmentation in B16F10 cells by downregulating the expression of these proteins. Eckol downregulated the expression of these melanogenic proteins in a similar fashion as previously reported [31], while downregulation by 974-A and phlorofucofuroeckol-A was evaluated for the first time.

### 3.8. Effect on the Intracellular ROS Level

Oxidative stress is the major cause of ROS generation, which damages deoxyribonucleic acid (DNA), proteins, and lipids in the body [34]. In addition, oxidative stress disrupts the homeostasis of melanocytes, eventually leading to malignancy or death [35]. Since melanin synthesis involves oxidation reactions and generates superoxide anion (O_2_^−^) and hydrogen peroxide (H_2_O_2_), it is important to inhibit the production of these reactive oxygen species to prevent hyperpigmentation.

**Figure 5:**
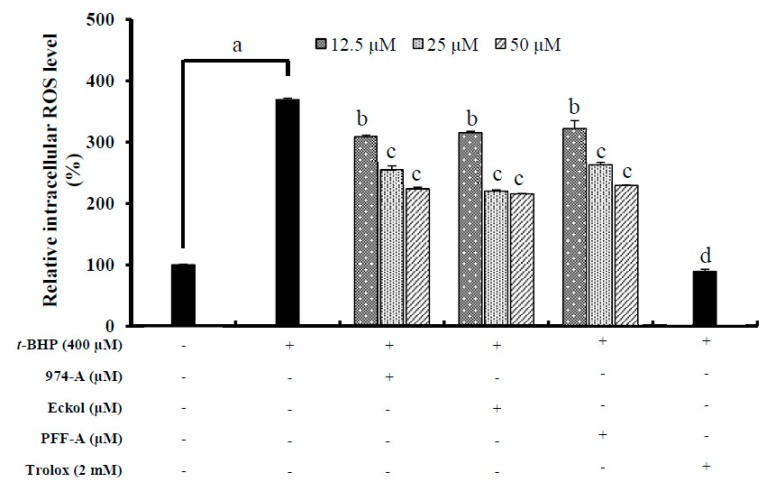
Effect of different concentrations of 974-A, eckol and phlorofucofuroeckol-A (PFF-A) on intracellular reactive oxygen species (ROS) levels in *tert*-butyl hydroperoxide (*t*-BHP) treated B16F10 melanoma cells. Cells were pretreated with the indicated concentration of 974-A, phlorofucofuroeckol-A, eckol along with *t*-BHP (400 µm) and trolox for 2 h, and then with 2′,7′-dichlorodihydrofluorescein diacetate (DCFH-DA) (25 µM) for 30 min to induce ROS generation. Data represent the mean ± SD of three independent experiments. ^a^ *p* < 0.01 indicates significant differences from the normal control group (no treatment); ^b^ *p* < 0.05, ^c^ *p* < 0.01, and ^d^ *p* < 0.001 indicate significant differences from the alpha-melanocyte-stimulating hormone (α-MSH) treated group.

To determine whether the isolated compounds reduced oxidative stress, we evaluated their effects on ROS generation in B16F10 melanoma cells treated with 400 μM *t*-BHP. ROS levels were greater in *t*-BHP-treated cells than in untreated cells (Figure 5). Pretreatment with the compounds at 12.5, 25, and 50 μM significantly inhibited ROS generation. The multifunctional antioxidant activities of polyphenols such as phlorotannins are highly related to their phenol rings, which act as electron traps to scavenge peroxynitrite (ONOO^−^), superoxide anions (O_2_^−^), and hydroxyl radicals (OH^−^). Although these compounds exhibited moderate scavenging abilities in comparison to the positive control, Trolox (2 mM), they still could scavenge ROS applied at concentrations as high as 50 μM.

## 4. Conclusions

Our study identified potent anti-tyrosinase compounds from the seaweed *E. stolonifera*. For the first time, 974-A was established as a potent and dose-dependent inhibitor of tyrosinase activity towards both of its substrates, as well as a radical scavenger. A molecular docking simulation revealed the importance of the hydroxyl moiety in exerting this anti-tyrosinase activity. Compound 974-A, together with other isolated compounds (phlorofucofuroeckol-A and eckol), reduced the melanin content and tyrosinase activity of cells, and downregulated the expression of melanogenic enzymes (tyrosinase, TRP-1, and TRP-2) in a dose-dependent manner. Consequently, compounds isolated from the marine seaweed *E. stolonifera* (974-A, phlorofucofuroeckol-A, and eckol) could be used as tyrosinase inhibitors and be further explored in the cosmetic and agricultural fields.

## Figures and Tables

**Table 1:** Mushroom tyrosinase inhibitory and free radical scavenging activity of phlorotannins isolated from *E. stolonifera* along with the kinetic analysis of 974-A.

Compounds	IC_50_ Value (µM, Mean ± SD) ^1^					Type of inhibition ^2^, *K_i_* Value ^3^	
	Mushroom Tyrosinase Inhibition		Radical Scavenging Activity				
	l-Tyrosine	l-DOPA	ONOO^−^	DPPH	ABTS^•+^	l-Tyrosine	l-DOPA
974-A	1.57 ± 0.08	3.56 ± 0.22	0.26 ± 0.06	0.92 ± 0.11	6.29 ± 0.34	Competitive, 0.69	Competitive, 3.33
Phlorofucofuroeckol-A	3.42 ± 0.01	8.14 ± 0.29	0.85 ± 0.10	1.92 ± 0.09	12.87 ± 0.95	Non-competitive, 2.70	Non-competitive, 7.14
Eckol	9.12 ± 0.36	29.59 ± 0.48	0.73 ± 0.07	1.63 ± 0.65	5.01 ± 0.19	Non-competitive, 8.60	Non-competitive, 28.47
Phloroglucinol	251.00 ± 5.52	>400	1.72 ± 0.59	>100	31.48 ± 1.68	-	-
Arbutin ^4^	172.82 ± 4.70	>500	-	-	-	-	-
Kojic acid ^4^	7.82 ± 0.70	9.35 ± 0.70	-	-	-	-	-
Penicillamine ^4^	-	-	1.07 ± 0.21	-	-	-	-
Ascorbic acid ^4^	-	-	-	0.69 ± 0.04	-	-	-
Trolox ^4^	-	-	-	-	18.40 ± 0.70	-	-

^1^ The 50% inhibition concentrations (IC_50_, µM) are expressed as the mean ± SD of triplicates. ^2^ Type of inhibition determined by Lineweaver-Burk plot. ^3^ Inhibition constant (*K_i_*) determined by Dixon plot. ^4^ Positive controls.

**Table 2:** Binding sites and docking scores of phlorotannins and reference compounds in oxy-form tyrosinase from *Agaricus bisporus*.

Compounds	Binding Energy (kcal/mol)	No. of H-Bonds	H-Bond Interaction Residues	Hydrophobic Interacting Residues	Others
974-A	−3.92	8	Asp191, Ser282, His285, Met280, Asn260, Glu189, Tyr65	Pi-Sigma: Val283, Pi-Pi T-shaped: Phe192, Phe264, Pi-Alkyl: Pro284, Val283, Pro284	Electrostatic bond: His285, Glu189, van der Waals: Cu401
Phlorofucofuroeckol-A	−8.79	8	Tyr78, Asn81, Arg321, Ala246, His85, Lys79, Ala323, Tyr65	Pi-Sigma: Ala80, Pi-Pi T-shaped: His85, Pi-Alkyl: Arg321	Electrostatic bond: His244, Glu322
Eckol	−6.29	6	His85, Cys83, Glu322, Tyr65, Ala323, Lys79	Pi-Sigma: Ala80, Pi-Alkyl: Pro284	-
Phloroglucinol	−4.87	5	Arg321, Gly86, Glu239, Val88, Met319	-	-
l-Tyrosine ^1^	−6.31	5	His244, Asn260, Met280, Glu256 (Salt-bridge)	Pi-Alkyl: Ala286, Pi-Sigma: Val283, Pi-Pi Stacked: His263	van der Waals: Cu401, Cu400, Per402
Luteolin ^1^	−6.19	4	Cys83, Gly245, Ala246, Val248	Pi-Alkyl: Val248, Pi-Sigma: His85	Electrostatic bond: Glu322

^1^l-Tyrosine and luteolin are used as positive control as competitive and allosteric inhibitor, respectively.

## Supplementary Materials

The following are available online at https://www.mdpi.com/2076-3921/8/8/240/s1. Figure S1: Concentration-dependent graph of tyrosinase inhibitory activity of 974-A (A), eckol (B), and phlorofucofuroeckol-A (C) along with standard kojic acid using l-tyrosine and l-DOPA as substrate; Figure S2: Lineweaver-Burk plots for the inhibition of tyrosinase by 974-A, eckol, and phlorofucofuroeckol-A with l-tyrosine (A–C) and l-DOPA as substrates (D–F), respectively; Figure S3: Dixon plots for the inhibition of tyrosinase by 974-A, eckol, and phlorofucofuroeckol-A with l-tyrosine (A–C) and l-DOPA as substrates (D–F) respectively; Figure S4: Effects of 974-A (I), phlorofucofuroeckol-A (II), and eckol (III) on cell viability of B16F10 melanoma cells (A) and co-treatment with 3 μM α-MSH (B).
