# Supplementary material for: Phlorotannins with Potential Anti-Tyrosinase and Antioxidant Activity Isolated from the Marine Seaweed Ecklonia stolonifera
Source: Antioxidants (Basel). 2019 Jul 24;8(8):240. doi: 10.3390/antiox8080240 (PMC6720259; doi:10.3390/antiox8080240)
Supplement: Supplementary file 1 [file antioxidants-08-00240-s001.pdf]

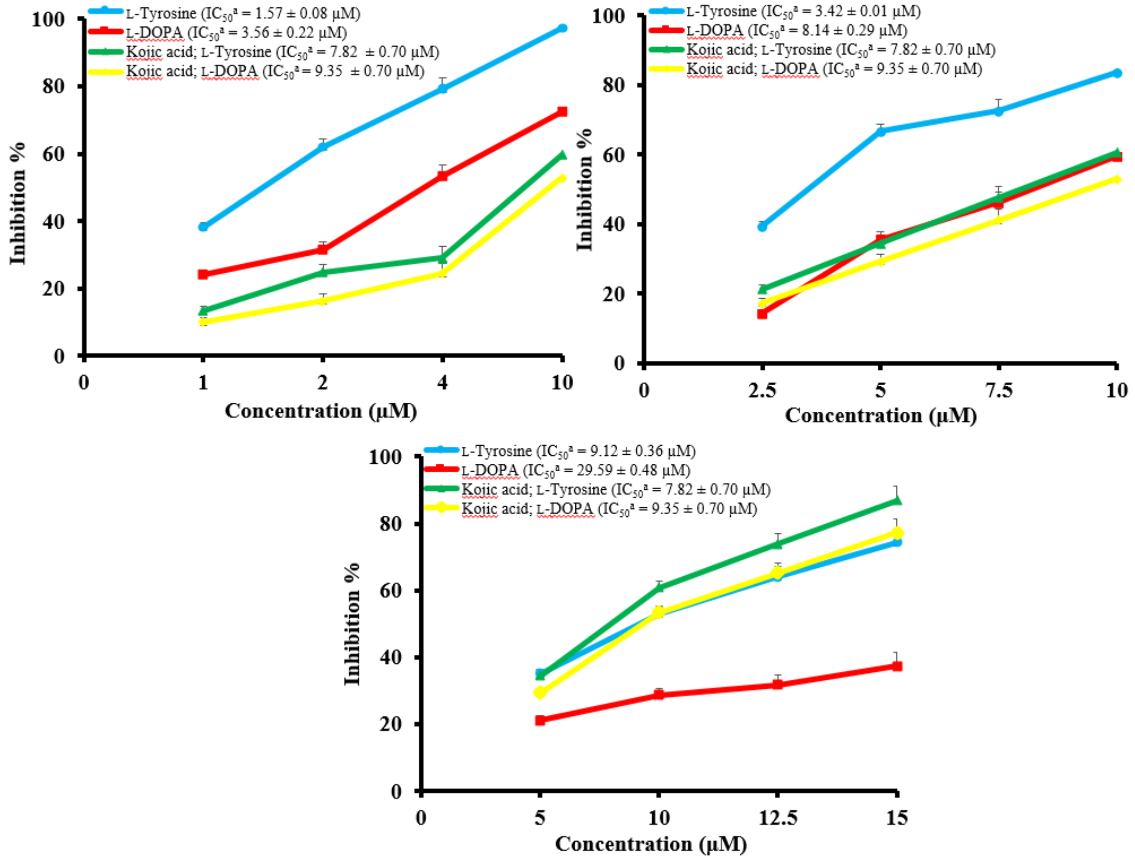

Figure S1: Concentration-dependent graph of tyrosinase inhibitory activity of 974-A(A), eckol (B) and phlorofucofuroeckol-A (C) along with standard kojic acid using L-tyrosine and L-DOPA as substrate. Error bars indicate standard deviation.

[a]The 50% inhibitory concentration (IC_{50}) values (μM) were calculated from a dose inhibition curve and expressed as mean ± SD of triplicates experiments

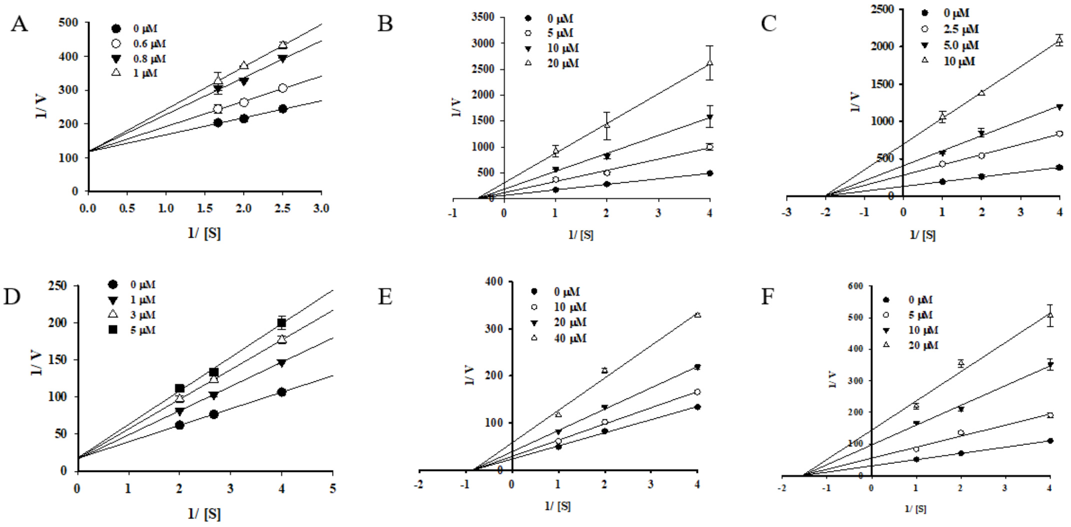

Figure S2: Lineweaver-Burk plots for the inhibition of tyrosinase by 974-A, eckol and phlorofucofuroeckol-A with L-tyrosine (A, B, and C) and L-DOPA as substrates (D, E and F) respectively

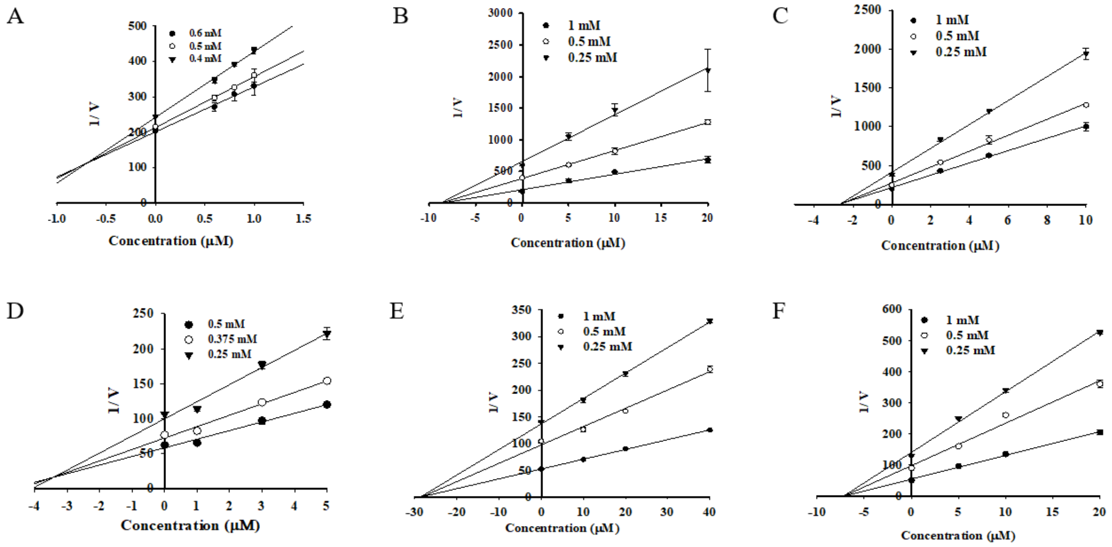

Figure S3: Dixon plots for the inhibition of tyrosinase by 974-A, eckol and phlorofucofuroeckol-A with L-tyrosine (A, B, and C) and L-DOPA as substrates (D, E and F) respectively

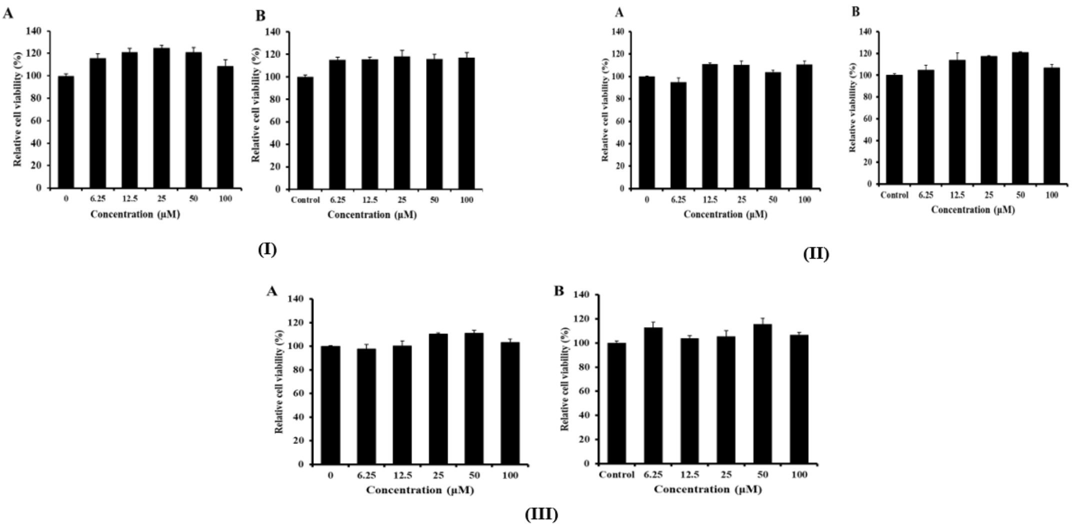

Figure S4: Effects of 974-A (I), phlorofucofuroeckol-A (II), and eckol (III) on cell viability of B16F10 melanoma cells (A) and co-treatment with 3 µM α-MSH (B).

Cell viability was determined using the 3-(4,5-dimethylthiazol-2-yl)-2,5-diphenyl tetrazolium bromide (MTT) method. Cells were pretreated with the indicated concentrations (6.25, 12.5, 25, 50, and 100 µM) of test compound for 48 h. Data show the mean ± SD of triplicate experiments
